# The Implementation of CDC COVID-19 Recommendations for Testing, Isolation, Quarantine and Movement at Emergency Intake Sites of Unaccompanied Children in the United States, April 1–May 31, 2021

**DOI:** 10.1007/s10903-023-01495-0

**Published:** 2023-06-14

**Authors:** Nirma D. Bustamante, Erin Sauber-Schatz, Deborah Lee, Kibrten Hailu, Yecai Liu, Clelia Pezzi, Joel Yonkman, Jose Gonzalez, Allen Appelgate, Nina Marano, Drew L. Posey, Martin Cetron, Edgar Monterroso

**Affiliations:** 1 Centers for Disease Control and Prevention (CDC), 1600 Clifton Rd, Atlanta, GA 30329 USA; 2 Administration for Children and Families (ACF), 330 C St. SW, Washington, DC 20201 USA; 3 Federal Emergency Management Agency (FEMA), 500 C St SW, Washington, DC 20024 USA

**Keywords:** Unaccompanied children, United States southwest border, COVID-19, SARS-CoV-2, Mitigation strategy, Congregate housing, Emergency intake sites

## Abstract

In March 2021, Emergency Intake Sites (EIS) were created to address capacity shortfalls during a surge of Unaccompanied Children at the Mexico-United States land border. The COVID-19 Zone Plan (ZP) was developed to decrease COVID-19 transmission. COVID-19 cumulative percent (%) positivity was analyzed to evaluate the impact of the ZP, venue type and bed capacity across EIS from April 1–May 31, 2021. Results: Of 11 EIS sites analyzed, 54% implemented the recommended ZP. The overall % positivity was 2.47% (95% CI 2.39–2.55). The % positivity at EIS with the ZP, 1.83% (95% CI 1.71–1.95), was lower than that at EIS without the ZP, 2.83%, ( 95% CI 2.72–2.93), and showed a lower 7-day moving average of % positivity. Conclusion: Results showed a possible effect of the ZP on % positivity when controlling for venue type and bed capacity in a specific EIS group comparison, indicating that all three variables could have had effect on % positivity. They also showed that smaller intake facilities may be recommendable during public health emergencies.

## Background

In March 2021, the surge in the number of Unaccompanied Children^1^ (UC) crossing the Mexico-United States (U.S.) border exceeded available shelter capacity, creating a humanitarian emergency [1–3]. Responsibilities for the care and placement of UC were transferred to the U.S. Department of Health and Human Services’ (DHHS) Director of the Office of Refugee Resettlement (ORR) from the U.S. Department of Homeland Security’s (DHS) Customs Border Protection (CBP) [4]. During periods of significant influx, ORR may operate temporary facilities called Emergency Intake Sites (EIS) or Influx Care facilities (ICF) to accommodate a surge in referrals if state-licensed care provider facilities are nearing full operating capacity [5]. Therefore, Emergency Intake Sites (EIS) were created to provide basic standards of care used for children in an emergency response setting [6]. ORR invited the U.S. Centers for Disease Control and Prevention (CDC) to provide technical assistance and site-specific recommendations to prevent the transmission of SARS-CoV-2 (the virus that causes COVID-19) among UC and site/facility staff. CDC developed and helped implement the COVID-19 Zone Plan (hereafter ZP) as well as recommended routine infection control measures. COVID-19% positivity (hereafter % positivity) was analyzed to evaluate the impact of the ZP in EIS.

## Methods

**Fig. 1:**
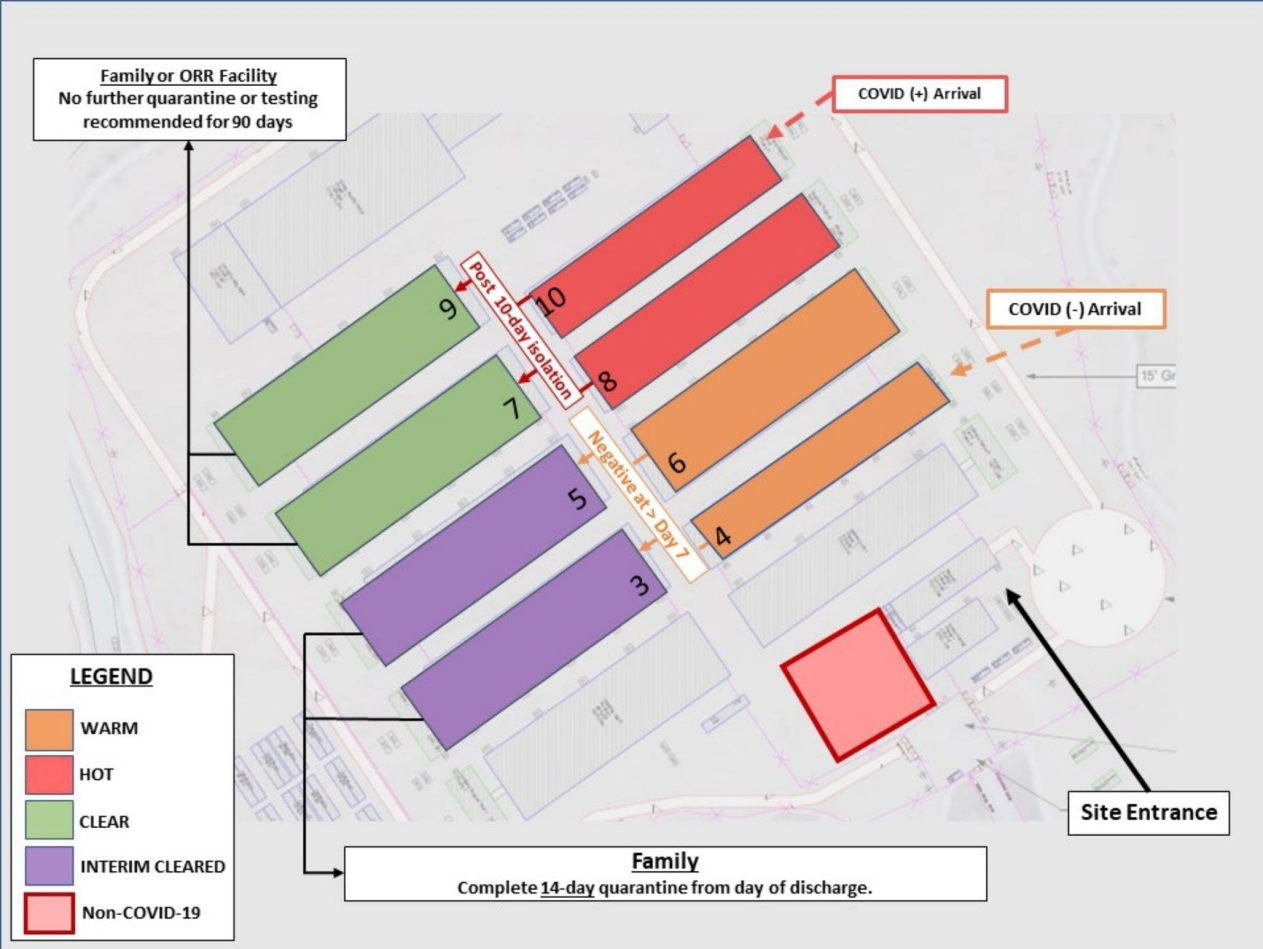
General depiction of the CDC-recommended COVID-19 Zone Plan implemented in Office of Refugee Resettlement (ORR) Emergency Intake Sites (EIS) for unaccompanied children (UC) during the COVID-19 pandemic^⁂^ ^⁂^ Newly arrived UC who tested negative before arrival were housed in the Warm Zone where they commenced the recommended 7-day testing algorithm, with testing upon arrival and every three days. UCs who tested positive before or upon arrival or at any point during their stay at the EIS, were transferred to the Hot Zone for a 10-day isolation period. UC who tested negative throughout the 7-day testing algorithm in the Warm Zone were transferred to the Interim Cleared Zone for the remainder of the EIS stay. The Clear Zone housed UC who were previously in the Hot Zone but completed a 10-day isolation and were not required to be tested or quarantined (if re-exposed) for 90 days. UCs with other communicable diseases were isolated in the non-COVID-19 Zone. UC could be transferred to a longer-term ORR facility from the Clear Zone or to a vetted sponsor from the Clear Zone or Interim Cleared Zone. If placed with a vetted sponsor for the Interim Cleared Zone, UC were recommended to complete a 14-day quarantine from day of discharge

The ZP (Fig. 1) was a 5-zone isolation, quarantine, and movement strategy for UC and incorporated a 7-day testing algorithm outlined in CDC’s “Interim Guidance for SARS-CoV-2 Testing in Correctional and Detention Facilities” [7]. The five zones included: (1) Warm Zone: UC who tested negative prior to being transferred to EIS, but who may have been exposed to COVID-19 during travel, and who commenced a 7-day testing algorithm with testing upon arrival then every three days; (2) Hot Zone: UC who tested positive before being transferred to or while at an EIS were isolated until 10 days after their date of symptom onset or date of positive test if asymptomatic; (3) Interim Cleared Zone: UC who tested negative throughout the 7-day testing algorithm in the Warm Zone, with continued repeat testing every three days for the duration of their stay due to the ongoing risk of exposure in the EIS; (4) Clear Zone: UC who were previously in the Hot Zone, but completed a 10-day isolation and, due to prior infection, did not require testing or quarantine (if re-exposed) for 90 days; and (5) Non-COVID-19 Zone: UC with other communicable diseases were isolated and continue the 7-day testing algorithm. Movement of UC was adjusted for each EIS. Access to shared facilities was limited to one zone at a time and cleaned before UC from another zone entered. Implementation of the ZP was elective. EIS that were not able to implement all zones described above were considered EIS that did not implement the ZP.

COVID-19 testing data (number of UC with positive result and total number of UC tested) were collected through daily internal situation reports at each EIS from April 1–May 31, 2021. EIS were established at convention centers, dormitories, and soft-sided facilities.^2^ For evaluation of bed capacity, EIS were categorized as having < 500, 500–1500, or > 1500 beds. Descriptive analysis compared % positivity of EIS by ZP implementation, venue type and bed capacity. Multivariate analysis could not be applied due to data limitations. EIS with the ZP did not include soft-sided facilities and those at convention centers did not have bed capacity < 500 or 500–1500. Additionally, EIS without the ZP at convention centers and dormitories did not have bed capacity of < 500 or 500–1500. Using comparative subsets of the data, % positivity for each of the three variables (the ZP, venue and bed capacity) was compared. The Chi-square test was used in comparisons with a p-value of < 0.05 for significance of difference. Given the observational nature of the limited data available, analysis could also not control the potential confounding factors of % positivity among staff, % positivity among UC upon arrival, and distribution of sex and age. Temporal trends in % positivity was assessed and confirmed using joint point regressions analysis.

UC could refuse testing, although refusal was rare. Informed consent for data analysis was not obtained because data were collected for programmatic purposes and did not include personally identifiable information.DHHS and DHS oversaw testing using rapid antigen tests per the 7-day testing algorithm. Only one result was accepted if a UC received multiple tests in one day. The routine aggregate data were provided to the emergency response team. Authors did not have direct interaction with UC for data collection. Data were stored securely and visualized through an internal dashboard at CDC. This evaluation was approved as non-research by a CDC human subjects’ adviser.

## Results

In total, 14 EIS were created across the U.S. Table 1 describes data for the 11 of the 14 EIS analyzed. Six (54%) implemented the ZP. Of these, four (67%) were in dormitory venues and two (33%) in convention centers; two (33%) had < 500 beds, three (50%) had 500–1500 beds, and one (17%) had > 1500 beds. Of the five EIS without the ZP, one (20%) was in a dormitory venue, one (20%) in a convention center, and three (60%) in soft-sided facilities; one (20%) had < 500 beds, one (20%) had 500–1500 beds, and three (60%) had > 1500 beds. Of the 11 EIS, four (36%) were male-only, six (54%) were mixed-sex, and one (9%) was female-only. Four (36%) EIS were designated for children younger than age 12 years. Three EIS were excluded from analysis due to missing data or had discordant data input, two dormitories and one soft-sided facility, which all had a bed capacity of 500–1500. One dormitory and soft-sided EIS had ZP implementation, and one dormitory did not have ZP implementation.

**Table 1:** Characteristics of emergency intake site (EIS) for unaccompanied children entering the United States at the southern land border, April 1–May 31, 2021

Site ID	COVID-19 Zone Plan implementation	Venue Type	Bed Capacity	Age of Unaccompanied Children	Sex of Unaccompanied Children	COVID-19 Percent Positivity (95% CI)
A	Yes	Dormitory	< 500	< 12 years old	Male only	1.39% (0.82,1.95)
H	Yes	Dormitory	< 500	< 18 years old	Male only	1.10% (0.79, 1.40)
L	Yes	Dormitory	500–1500	< 12 years old	Male and Female	0.81% (0.55, 1.07)
I	Yes	Convention Center	500–1500	< 12 years old	Female only	2.01% (1.61, 2.40)
N	Yes	Convention Center	500–1500	< 12 years old	Male and Female	2.71% (2.46, 2.96)
M	Yes	Dormitory	> 1500	< 18 years old	Male only	1.48% (1.31, 1.66)
D	No	Soft-sided facility	< 500	< 18 years old	Male and Female	1.14% (0.82,1.46)
C	No	Soft-sided Facility	500–1500	< 18 years old	Male and Female	1.54% (1.33, 1.76)
K	No	Dormitory	> 1500	< 18 years old	Male and Female	1.96% (1.72, 2.19)
B	No	Convention Center	> 1500	< 18 years old	Male only	1.38% (1.21,1.56)
F	No	Soft-sided facility	> 1500	< 18 years old	Male and Female	4.21% (4.03,4.40)

CI = confidence interval

Table 2 shows the descriptive and comparison analysis of % positivity. The overall % positivity across all EIS was 2.47% (95% Confidence Interval (CI) 2.39–2.55). The % positivity at EIS with the ZP, 1.83% (95% Confidence Interval (CI) 1.71–1.95) was lower than that at EIS without the ZP, 2.83% (95% CI 2.72–2.93). The % positivity was 1.51% (95% CI 1.40–1.63), 2.02% (95% CI 1.88–2.16) and 3.44% (95% CI 3.29–3.59) at EIS of venue of dormitory, convention center and soft-sided facility, respectively. The % positivity was 1.16% (95% CI 0.96–1.37), 2.01% (95% CI 1.81–2.15) and 2.81% (95% CI 2.70–2.91) at EIS of bed capacity of < 500, 500–1500 and > 1500, respectively.

**Table 2:** Descriptive and comparative analysis of COVID-19% positivity (% positivity) among unaccompanied children at office of refugee resettlement emergency intake sites (EIS) from April 1–May 31, 2021

	% Positivity (95% Confidence Interval)
**Overall**	2.47% (2.39, 2.55)
With ZP	1.83% (1.71, 1.95)
Without ZP	**2.83% (2.72, 2.93)**
**By Venue Type**	
Dormitory	1.51% (1.40, 1.63)
Convention Center	2.02% (1.88, 2.16)
Soft-sided facility	**3.44% (3.29, 3.59)**
**By Bed Capacity**	
< 500 beds	1.16% (0.96, 1.37)
500–1500 beds	2.01% (1.87, 2.15)
> 1500 beds	**2.81% (2.70, 2.91)**
**Comparati** **ve analysis** ^a^	
Controlling for EIS in dormitory venue type and > 1500 beds	
With ZP	1.48% (1.31, 1.66)
Without ZP	**1.96% (1.72, 2.19)**
Controlling for EIS with ZP and dormitory venue type	
< 500 beds	1.18% (0.91, 1.45)
500–1500 beds	0.81% (0.55, 1.07)
>1500 beds	**1.48% (1.31, 1.66)**
Controlling for EIS without ZP and > 1500 beds	
Convention Center	1.38% (1.21, 1.56)
Dormitory	1.96% (1.72, 2.19)
Soft-Sided Facility	**4.21% (4.03, 4.40)**
Controlling for EIS without ZP and soft-sided facility venue type	
< 500 beds	1.14% (0.82, 1.46)
500–1500 beds	1.54% (1.33, 1.76)
> 1500 beds	**4.21% (4.03, 4.40)**

^a^ Significance was set at p < 0.05. Chi-square test for all comparative analysis described was within set p-value.

Comparative analysis describes % positivity within EIS group variations by controlling for two of the three variables. For EIS in a dormitory venue and with > 1500 beds, those without a ZP had a higher % positivity, 1.96% (95% CI 1.72–2.19), compared to those without a ZP, 1.48%, (95% CI 1.31–1.66). Among EIS with ZP and in a dormitory, % positivity was 1.18% (95% CI 0.96–1.45), 0.81% (95% CI 0.55–1.07), and 1.48% (95% CI 1.31–1.66) for bed capacity of < 500, 500–1500, and > 1500, respectively. Among EIS without ZP and with bed capacity of > 1500, % positivity was 1.38% (95% CI 1.21–1.56), 1.96% (95% CI 1.72–2.19), and 4.21% (95% CI 4.03–4.40) for convention center, dormitory, and soft-sided facility, respectively. Among EIS without ZP and at soft-sided facilities, % positivity was 1.14% (95% CI 0.82–1.46), 1.54% (95% CI 1.33–1.76), and 4.21% (95% CI 4.03–4.40) for a bed capacity of < 500, 500–1500, and > 1500, respectively.

**Fig. 2:**
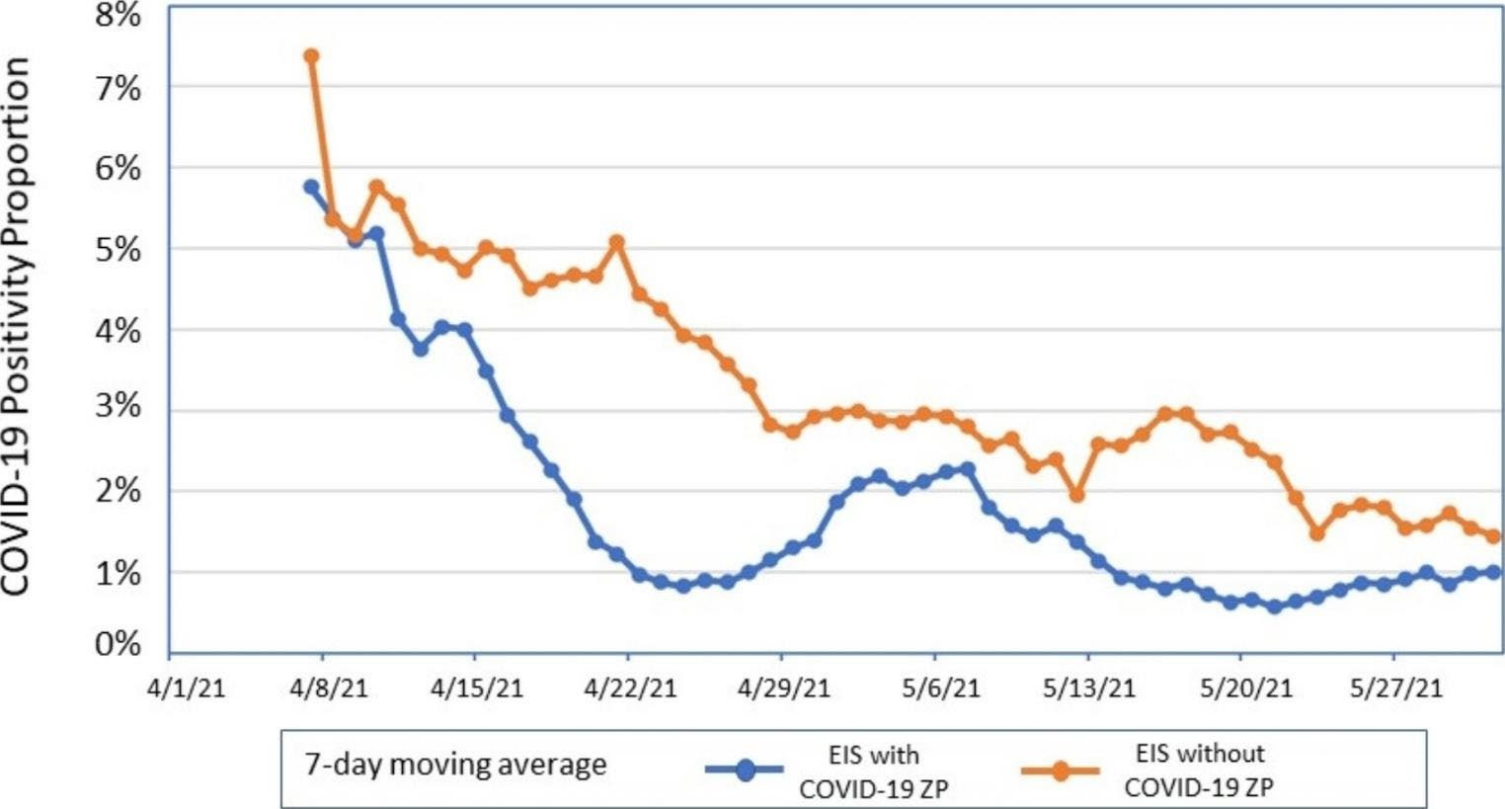
Seven-day moving average of COVID-19% positivity comparing Office of Refugee Resettlement Emergency Intake Sites (EIS) for Unaccompanied Children that implemented or did not implement the COVID-19 Zone Plan, April 1-May 31, 2021

The temporal trend of the 7-day moving average of % positivity was also evaluated. EIS using the ZP showed a lower % positivity over the analysis period (Fig. 2). Joint point regression analysis confirmed results (not shown).^4^

## Discussion

Due to the observational nature of the analysis and confounding factors, the direct impact of COVID-19 ZP on % positivity could not be evaluated. The uneven implementation of the ZP among site type and bed capacity was affected by facility leadership preferences and unique logistical limitations at each EIS. This was clear with the distribution of EIS with the ZP limited to dormitories and convention centers and 60% of EIS without the ZP at soft-sided facilities and with a bed capacity > 1500. Data collection during humanitarian emergencies is often difficult due to conditions inherent in the response and may limit the strength of the data [8]. Similar evaluations of COVID-19 testing, isolation, quarantine, and movement strategies are sparse. The literature is limited to recommendations based on best practices, qualitative evaluations, and mathematical modeling [9–10]. Albeit observational, findings presented here are based on quantitative analysis and are an example of a collaboration between CDC, ORR, and FEMA, which also highlights the importance of interagency coordination during a humanitarian emergency.

EIS did not have comparable sex and age distributions; therefore, these variables were not included in comparative analysis. In addition, EIS were set by bed capacity; thus, overcrowding was not assessed. Results suggest that implementation of a ZP could be of value in responding to COVID-19 in certain settings with strong consideration to venue and bed capacity dynamics. This is exemplified by the higher % positivity among EIS without a ZP while controlling for a dormitory venue type and > 1500 bed capacity. As referenced above, % positivity among EIS without the ZP was highest among soft-sided facilities and > 1500 beds. Specifically, site F, which had the largest bed capacity among all EIS (7,200 beds) and could be a factor skewing data among these sites. Based on results, it may be recommendable to only use smaller intake facilities to minimize risk of exposure in congregate settings during public health emergencies.

CDC provided interim guidance to reduce the risk of COVID-19 spread which included but not limited to wearing masks, COVID-19 screening prior to entering EIS, COVID-19 vaccination, routine testing, prevention materials, handwashing stations, cleaning, physical distance, and quarantine/isolation. Adherence to these factors may have impacted % positivity, but data were not available to evaluate additional confounding.

Results are specific to emergency shelters in the United States, where infrastructure and resources were available. Unique challenges at specific emergency shelters need to be taken into consideration in locations where security, sanitation and hygiene, or nutritional provisions are not guaranteed. Despite limitations, the ZP could be considered as a field mitigation strategy during a public health emergency in a humanitarian emergency. Because it was not possible to control confounding factors (i.e., venue and bed capacity), further studies are needed to assess the association between % positivity and the implementation of the ZP.

## Data Availability

The datasets generated and/or analyzed during the current study are not publicly available for the protection of unaccompanied minors.

## Footnotes

1 Unaccompanied Child (UC) — A UC is defined as a child who has no lawful immigration status in the United States; has not attained 18 years of age; and, with respect to whom, there is no parent or legal guardian in the United States, or no parent or legal guardian in the United States available to provide care and physical custody. See 6 U.S.C. § 279(g) [2].

2 Convention Center—temperature-regulated facility with single cots in a large open space. Dormitory – facility with individual rooms accommodating one or two persons per room. Soft-sided facility—temporary, standalone, industrial-size tent powered by electricity supply and with heating, ventilation, and air conditioning (HVAC) system, accommodating single cots in a large open space. Convention center and dormitory were established structures and not soft-sided.

4 Available upon request.
